# Antibiotic Exposure and Other Risk Factors for Antimicrobial Resistance in Nasal Commensal *Staphylococcus aureus*: An Ecological Study in 8 European Countries

**DOI:** 10.1371/journal.pone.0135094

**Published:** 2015-08-11

**Authors:** Evelien M. E. van Bijnen, John Paget, Elly S. M. de Lange-de Klerk, Casper D. J. den Heijer, Ann Versporten, Ellen E. Stobberingh, Herman Goossens, François G. Schellevis, collaboration with the APRES Study Team

**Affiliations:** 1 NIVEL, Netherlands Institute for Health Services Research, Utrecht, The Netherlands; 2 VU University Medical Centre, Department of Epidemiology and Biostatistics, Amsterdam, The Netherlands; 3 Maastricht University Medical Centre, Department of Medical Microbiology, School for Public Health and Primary Care (CAPHRI), Maastricht, The Netherlands; 4 University of Antwerp, Laboratory of Medical Microbiology, Vaccine & Infectious Disease Institute (VAXINFECTIO), Antwerp, Belgium; 5 VU University Medical Centre, Department of General Practice and Elderly Care Medicine/EMGO Institute for Health and Care Research, Amsterdam, The Netherlands; Institut National de la Recherche Agronomique, FRANCE

## Abstract

**Objectives:**

Antimicrobial resistance (AMR) has become a global public health concern which threatens the effective treatment of bacterial infections. Resistant *Staphylococcus aureus* (including MRSA) increasingly appears in individuals with no healthcare associated risks. Our study assessed risk factors for nasal carriage of resistant *S*. *aureus* in a multinational, healthy, community-based population, including ecological exposure to antibiotics.

**Methods:**

Data were collected in eight European countries (Austria, Belgium, Croatia, France, Hungary, the Netherlands, Spain and Sweden). Commensal AMR patterns were assessed by collecting 28,929 nasal swabs from healthy persons (aged 4+). Ecological exposure to antibiotics was operationalized as systemic antibiotic treatment patterns, extracted from electronic medical records of primary care practices in which the participants were listed (10–27 per country). A multilevel analysis related AMR in nasal commensal *S*. *aureus* to antibiotic exposure and other risk factors (e.g. age and profession).

**Results:**

Of the 6,093 *S*. *aureus* isolates, 77% showed resistance to at least one antibiotic. 7.1% exhibited multidrug resistance (defined as resistance to 3 or more antibiotic classes), and we found 78 cases MRSA (1.3%). A large variation in antibiotic exposure was found between and within countries. Younger age and a higher proportion of penicillin prescriptions in a practice were associated with higher odds for carriage of a resistant *S*. *aureus*. Also, we found higher multidrug resistance rates in participants working in healthcare or nurseries.

**Conclusions:**

This study indicates that in a population with no recent antibiotic use, the prescription behavior of the general practitioner affects the odds for carriage of a resistant *S*. *aureus*, highlighting the need for cautious prescribing in primary care. Finally, since variation in AMR could partly be explained on a national level, policy initiatives to decrease AMR should be encouraged at the national level within Europe.

## Introduction

Increasing antimicrobial resistance (AMR) has become a global public health problem in recent decades, threatening effective treatment of bacterial infections [1–4]. Whilst scientific interest and available AMR data in the previous century was mainly focussed on hospital settings, the last decade has seen an increase in interest in community-associated resistance [5–7]. Traditionally, the occurrence of resistant *Staphylococcus aureus* (including methicillin resistant *S*. *aureus*, MRSA) was confined to hospitals and long-term-care facilities. In the 21th century however, MRSA infections have also appeared in community-dwelling individuals with no healthcare associated risks such as a recent hospitalization [8–11]. Most bacterial infections are caused by the patients’ own commensal microbiota [12,13], which forms a reservoir of bacterial antibiotic resistance genes [14,15]. *S*. *aureus* is a commensal pathogen mainly associated with bacterial skin and soft tissue infections (SSTIs) and sometimes with pneumonia [6,13,16]. The incidence of these infections in primary care is relatively high, thereby representing a frequent indication for antibiotic treatment [16,17].

Administration of antibiotics is associated with resistance and 90% of all antibiotics are prescribed in primary care [18–20]. Several studies have advocated cautious and appropriate treatment with antibiotics in primary care [21,22]. Ineffective and inappropriate antibiotic treatment has negative consequences for both the patient and the healthcare system: the infection remains untreated, the patient potentially suffers additional side-effects, AMR could develop due to the exposure to antibiotics and finally, unnecessary costs are made [23,24]. Nonetheless, still a wide variation in the rate of antibiotic treatments exists between countries, suggesting possible overtreatment [18,25]. Optimal empirical treatment in primary care should take into account AMR information from community-based patients [26]. However, AMR patterns vary widely across health care settings and across Europe [27], and information on relevant AMR patterns is often lacking in treatment guidelines, which could hamper the General Practitioner’s (GPs) choice for an effective antibiotic [28].

Part of the worldwide approach to control the spread of community-associated AMR consists of studies assessing risk factors for community-associated resistant *S*. *aureus* [5,13,29]. However, such studies often focus on isolated pathogenic bacteria and disregard the impact of exposure to antibiotics [30]. On the other hand, studies supporting the association between antibiotic use and AMR often concern streptococci or *E*. *coli*, use data on a national level or focus on local enclosed environments [29,31–35].

Our ecological study integrates current knowledge and investigates the nasal commensal microbiota, a source of bacterial infections [8,9], in the light of the relationship between antibiotic exposure and AMR [18–20]. Using ecological data on antibiotic exposure, and AMR patterns of nasal *S*. *aureus* in 8 European countries, we assess which factors are related to resistance in commensal nasal *S*. *aureus* in a community population with no health-care associated risks.

## Methods

### Data Collection

This study is part of the multinational APRES study, in which data has been collected on AMR and antibiotic treatment in primary care across Europe. Based on their varying volume of prescribed antibiotics [18,23], nine countries across Europe have participated in this collaborative study: Austria, Belgium, Croatia, France, Hungary, the Netherlands, Spain, Sweden, and the United Kingdom. A detailed overview of the APRES study design has been published elsewhere [36]. In this manuscript, we have assessed in a healthy community based population which factors are related to carriage of a resistant *S*. *aureus*, taking into account ecological antibiotic exposure. Data from eight countries have been used since the UK could not deliver antibiotic treatment data at substance level.

#### Ethics statement

Ethical approval for this study has been obtained in each of the participating countries, from the following ethics committees:

Austria: Ethik-Kommission der Medizinischen Universität Wien und des Allgemeinen Krankenhauses der Stadt Wien Akh (number: 568/2010)
Belgium: Commissie Medische Ethiek van de Universitaire Ziekenhuizen K.U.Leuven (number: ML6355)
Croatia: Sveučilišta u Zagrebu Medicinski Fakultet Ethical Committee (number: 04-77/2010-246)
France: Comité de protection des personnes CPP “Ile-de-France III” (number: 2010-A01004-35 (2853))
Hungary: Egészségügyi Tudományos Tanács, Tudományos es Kutatásetikai Bizottság (ETT TUKEB) (number:5635-0/2010-1018EKU (401/PI/010)
The Netherlands: Medisch Ethische Commissie azM/UM (number: MEC 10-4-030.4/pl)
Spain: Clinical Ethics Committee of the IDIAP Jordi Gol and Gurina (number: P10/55)
Sweden: Regionala Etikprövningsnämnden i Linköping (number: 2010-326-31)

All participants were informed and signed an informed consent form. For children consent was obtained from one of the parents or guardians.

#### AMR

To measure AMR in the nasal commensal flora in each of the countries, a targeted number of 4,000 nasal swabs were collected from healthy persons (aged 4+), visiting their primary care practice during the winter of 2010–2011. Per country, 17–27 practices participated, recruiting up to 200 persons (aged 4+) per practice. All participants received information and signed an informed consent form. For children, consent from one of their parents or their guardian is obtained. The participating practices were members of national General Practitioner Networks, and representative for GPs in their country. All eligible persons were invited in order of visiting the practice; however we aimed for stratification regarding gender (50% male, 50% female) and age (one third under 18, one third between 18 and 64, one third 65+ years old) during data collection. Persons with antibiotic use or hospitalization in the past 3 months, and persons with current infectious diseases were excluded from participation in order to assess the nasal commensal AMR level. National laboratories isolated *S*. *aureus* from the nasal swabs using standardized procedures. All isolated *S*. *aureus* were tested in a central laboratory (Maastricht University, the Netherlands) for susceptibility to 12 antibiotics assumed to represent a range of commonly used antibiotic classes (see S1 Table)) [37]: tetracycline, beta-lactamase susceptible penicillin, oxacillin, co-trimoxazole, erythromycin, azithromycin, clindamycin, gentamicin, ciprofloxacin, vancomycin, daptomycin and linezolid. The procedure included standardised micro dilution tests and classification (resistant versus susceptible) was based on the EUCAST guidelines [27,38]. For each antibiotic national resistance rates were calculated by aggregating the individual data.

Data were obtained from swabs from a total number of 28,929 persons: in twenty-one percent (N = 6,137) a *S*. *aureus* was isolated. Excluding participants without AMR data (0,7%) resulted in a total number of 6,093 individuals in our study. This sample consisted of 5,224 (85,7%) adults (aged 18+) and 839 (13,8%) children (aged 4 to 17), and 30 persons (0,5%) whose age was unknown. 51% of all participants was female.

We assessed nasal carriage of *S*. *aureus*, and categorized each microbe in two different ways:

Whether the isolated microbe is resistant at all:

1. Resistance to none of the tested antibiotics versus resistance to at least one the tested antibiotics.
  Multidrug resistance:
2. Resistance to 0, 1 or 2 antibiotic classes versus resistance to 3 or more antibiotic classes (ATC 3 level).

We included multidrug resistance as a variable instead of MRSA, since in our study only 78 MRSA isolates were found. They have been described in more detail elsewhere [27]. Previous studies [6] have shown carriage of *S*. *aureus* to be dynamic and occurring on multiple bodily sites. While the prevalence might be underestimated by using nasal swabs [27], we assume our final sample of *S*. *aureus* to be representative of all carriage.

#### Antibiotic exposure

Ecological antibiotic exposure was operationalized as antibiotic treatment volume and pattern of the primary care practices in which the participant was listed. We assume that the number of antibiotic prescriptions per practice is an approximation of exposure for the participants from that practice. Data about systemic antibiotic treatments (ATC codes J01) were extracted from the electronic medical records of the participating primary care practices from which this was possible (90%). Given that AMR developed after exposure to an antibiotic can linger up to one year [19], we included all antibiotic prescriptions of the calendar year 2010. As unit of measurement, we used packages, which were the common measure in the different datasets. The number of antibiotic packages per practice was aggregated at substance level according to the WHO Anatomical Therapeutic Chemical (ATC) classification system (ATC 5 level, 7 digit-code) [39]. For this paper we aggregated antibiotic package data at ATC5 level for ages >4 year.

To assess the association between antibiotic exposure and AMR we operationalized this exposure in two different ways:

1. The load of antibiotics is defined as the total number of packages/100 active patients prescribed by practice in 2010. Active patients are defined as those who have visited the practice at least once during the year 2010.
2. Secondly, we calculated the percentage of these prescribed packages which consists of beta-lactamase susceptible penicillin (since this is known to be the antibiotic with the highest resistance levels in *S*. *aureus* in the community [27]).

#### Other risk factors

All participants completed a short questionnaire with background information. Based on previous literature, we selected several variables which were used in our model. On the patient level, several variables have been demonstrated to increase the odds of AMR: a younger age [32], living with children [13], working with children [33], working in health care [40], working in the veterinary sector [41,42]. Next to this, chronic skin conditions are related to carriage of *S*. *aureus*, and we also included gender and the number of GP visits in the last year, since in all participating countries a prescription is needed for an antibiotic. We found no previous evidence for variables on practice level to be correlated to carriage of a resistant *S*. *aureus*.

### Data Analysis

The dependent variable (AMR) was nested on three levels: person, primary care practice, and country. A multilevel logit analysis was conducted (using MLwiN version 2.33), relating AMR to ecological antibiotic exposure, controlling for other risk factors. Both analyses were based on a dichotomous dependent variable: for model 1, it was coded 0 if an isolate shows no resistance and 1 if resistance to at least 1 antibiotic is found. For model 2, the response variable was coded 1 if an isolate is demonstrated to be multidrug resistant (otherwise coded 0). For useful interpretation of the results, the continuous variables in the analysis (age of the participant, total number of packages and percentage of penicillin prescriptions) were transformed into quartile scores (see S2 Table).

## Results

### AMR

The susceptibility tests showed overall low AMR levels in commensal nasal *S*. *aureus* in the community: a resistance rate of <20% to all antibiotics was found, except for (beta-lactamase susceptible) penicillin, to which 78% resistance was demonstrated (see S3 Table). In Table 1, it is shown that approximately 4 out of 5 isolates were resistant to at least one antibiotic (mostly penicillin), while 7.1% of the isolates were multidrug resistant (resistant to at least 3 antibiotic classes on ATC3 level).

**Table 1:** Percentages of AMR and antibiotic exposure levels per country.

Country (number of analysed isolates)	% AMR > 0 (range on practice level)	% multidrug resistance	% MRSA	Antibiotic prescriptions / 100 active patients* (range on practice level)	% of penicillin prescriptions (range on practice level)
**Austria (549)**	70 (54–92)	9.5	1.5	24.2 (7.6–45.9)	21.1 (3.4–65.8)
**Belgium (582)**	80 (67–100)	9.6	2.1	27.0 (7.2–61.8)	33.5 (8.0–61.7)
**Croatia (755)**	80 (67–90)	5.4	2.0	66.2 (33.7–112.5)	19.0 (6.6–39.6)
**France (874)**	81 (67–95)	11.0	1.8	18.8 (3.0–37.4)	36.8 (16.4–64.2)
**Hungary (539)**	81 (65–95)	10.2	1.5	68.1 (1.3–162.9)	20.2 (2.4–55.0)
**Netherlands (1073)**	71 (59–82)	4.1	0.8	30.9 (19.9–51.5)	24.7 (10.9–39.7)
**Spain (766)**	89 (76–100)	10	1.3	68.7 (41.9–184.6)	34.2 (24.4–45.2)
**Sweden (955)**	67 (55–76)	1.2	0	17.7 (8.4–33.2)	45.7 (33.0–53.3)

*Antibiotic prescriptions and denominator include data for persons > 4 years

### Antibiotic Exposure

In total, systemic antibiotic prescription data from 164 practices, ranging from 10 to 27 practices per country, could be analysed. This accounted for a total of 1,024,064 active patients (range 33,237–637,999 patients per country) (see S4 Table)). Antibiotics belonging to the penicillin group were the most often prescribed antibiotic, followed by macrolides, while linezolid, vancomycin and daptomycin were very rarely prescribed. In the analyses we included only those antibiotic classes that were included in the susceptibility tests; this is 80% of the prescribed antibiotics.

A large variation between countries was found: practices in Sweden and France stood out with a low number (<20) of prescribed packages per 100 active patients, whilst practices in Croatia, Hungary and Spain on average prescribed over 60 packages for every 100 active patients. Finally, the extent to which GPs opted for penicillin to treat bacterial infections differed: from 1 in every 5 prescribed packages in Croatia, to almost half of the prescriptions in Sweden. At the same time however, Sweden displayed the lowest antibiotic load, and the lowest AMR levels of all countries. Spain on the other hand demonstrated high AMR levels and a high average exposure to antibiotics (Table 1).

### Risk Factors for Carriage of Resistant *S*. *aureus*

Descriptive statistics of all variables are shown in S1 Table. The estimated odds of the final models are described in Table 2. Due to missing data on the explanatory variables, not all samples were included in the multilevel analysis.

**Table 2:** Independent risk factors for antibiotic resistance and for multidrug resistance (N = 5,191 *S*. *aureus* isolates).

Risk factors	Model 1: AMR		Model 2: Multidrug resistance	
	No resistance (0) versus resistance to at least one antibiotic (1)		Resistance to 0–2 antibiotic classes (0) versus multidrug resistance (1)	
	OR	95% C.I.	OR	95% C.I.
*Risk factors*				
Age Patient (quartile 1 = ref category)	0.88*	0.82–0.94	0.96	0.86–1.06
Gender Patient (male = ref category)	0.97	0.85–1.11	1.21	0.97–1.50
Number of GP visits (0 visits = ref category)	1.13	1.00–1.28	1.20	0.99–1.45
Work: Nursery	0.84	0.54–1.29	1.87*	1.07–3.26
Work: Health care	1.03	0.78–1.37	1.72*	1.14–2.60
Work: Livestock	1.08	0.70–1.66	1.30	0.70–2.43
Living with children (no = ref cat)	1.18	0.96–1.45	1.03	0.76–1.40
Skin condition	1.0	0.79–1.26	0.89	0.59–1.36
Prescriptions Total (quartile 1 = ref category)	1.04	0.94–1.15	1.13	0.98–1.30
% Penicillin (quartile 1 = ref category)	1.09*	1.00–1.18	1.0	0.90–1.11
*Random effect*				
Country level variance (SE)	0.148 (0.08)		0.324 (0.177)	
Practice level variance (SE)	0.034 (0.024)		0.008 (0.049)	
*Intercept*	1.168 (0.241)		-3.225 (0.355)	

*significant p<0.05

The first model assessed risk factors for carriage of a *S*. *aureus* resistant to at least one antibiotic. The results showed that participants with a younger age had a higher risk to carry a resistant *S*. *aureus*. Next to this, persons listed in practices with higher proportional penicillin exposure had a higher risk to carry a resistant *S*. *aureus*. Both results show relatively small odds, and are based on quartile scores for which a linear relationship was assumed. Overall, in the first model, almost no variation was found on the practice level, but a considerable variation was seen at the country level.

Results concerning multidrug resistance (Model 2) showed that certain professions are associated with multidrug resistance: participants working in health care or nurseries had a 1.7–1.9 times higher risk for carrying a multidrug resistant *S*. *aureus*. On the other hand, although penicillin prescriptions were related to AMR in general (model 1), the level of exposure to antibiotics had no significant effect on colonization of multidrug resistant bacteria.

## Discussion

Our study integrates and expands current knowledge by investigating the nasal commensal microbiota, which are a reservoir for resistant pathogens [8,9], taking into account the relationship between antibiotic exposure and AMR that has been demonstrated in pathogenic bacteria [18–20]. In two models, we assessed the risk factors for nasal carriage of a resistant *S*. *aureus* in a community population with no health-care associated risks. We identified several risk factors associated with carriage of a resistant *S*. *aureus*: age, profession, treatment with penicillin and country.

Our results show that resistance to any antibiotic is associated with lower age. Previous studies support this result [32]. We found higher resistance rates in practices with a high proportion of penicillin prescriptions, suggesting that the relationship between antibiotic exposure and antibiotic resistance also holds for the commensal nasal microbiota. At the same time however, Sweden, the country with the highest proportion of penicillin treatments, had the lowest total antibiotic exposure and displays the lowest AMR levels. This might be explained by different antibiotic prescription policies [43,44]. Sweden was one of the first countries to have developed a resistance surveillance network in the mid-nineties, together with Denmark and The Netherlands (respectively STRAMA, Danmap and SWAB [44–46]). These countries are known internationally for their low AMR rates both in hospitals and the community [5]. The activities of these networks are focussed on surveillance, research and policies of AMR in different settings. This is also congruent with our overall finding that variation is mostly found at the country level and not at the practice level.

Regarding multidrug resistance, we found that working in health care or a nursery is associated with multidrug resistance. Finally, most variance in resistance is found at the country level. This implies that although AMR is associated with antibiotic exposure in the GP practice, commensal AMR can be considered a national phenomenon. The preferred level of action should therefore be national [1]: already several initiatives have been developed throughout Europe, decreasing the level of AMR.

### Strengths and Limitations

A strength of this study is the integration of knowledge regarding community-associated *S*. *aureus*, and the broad perspective by including data from 8 European countries and focussing on a population without health-care associated risks. Within these countries, extensive prescription data and AMR data were collected, enabling unique analyses.

The study is limited regarding several assumptions that have been made in our analysis: although carriage of *S*. *aureus* is dynamic [6], we assume that our point-prevalence measure provides a good approximation of AMR in the commensal nasal flora for *S*. *aureus*. We assume that AMR patterns in the nasal commensal flora are comparable with those of pathogenic *S*. *aureus*. However, for empirical treatment purposes, AMR data on pathogenic isolates is also needed. Furthermore, we did not use antibiotic treatment data on patient level and used practice level antibiotic prescription data as an approximation of ecological exposure to antibiotics at the individual level. The healthcare system of the country could affect the representativeness of our measure of exposure to antibiotics. In some countries GPs function as a gatekeeper to the healthcare system, while elsewhere other medical disciplines also prescribe antibiotics. Although over the counter sales of antibiotics without prescriptions is prohibited, we cannot rule out the possibility of our patients being exposed to antibiotics elsewhere. Studies indicating major effects from other sources are lacking, therefore we assume that our measurement is a good approximation of the ecological exposure to antibiotics in the community, with a possible underestimation.

Our data collection of antibiotic prescriptions showed the difficulties of establishing a congruent database given the different ways of collecting treatment data across Europe (data was extracted from different electronic systems based on different classification models). Not all participating practices were able to deliver complete treatment data, which could indicate a selection bias towards more involved GPs. We assume the participating practices to be representative for the whole country regarding prescription behaviour. A comparison with ESAC reports [5] supports this assumption: the two data sources show comparable proportions and rates of antibiotic prescription, except for France. The patterns of antibiotic prescriptions do not differ, but in our study France reported lower antibiotic prescription rates compared with what is reported in ESAC, which indicates a lower representativeness of the participating practices in France. However, an analysis excluding the French data produced the same results.

### Implications

Our results demonstrated several risk factors for AMR in nasal commensal *S*. *aureus*; this should create a higher awareness under general practitioners. AMR can also spread to patients who are not directly exposed: in this ecological dilemma the benefits for the patient have to be weighed against the drawbacks for the total population. After correcting for other risk factors, we found mainly national variation in resistant *S*. *aureus*, indicating that national actions are recommended.

## Supporting Information

S1 Fig Antibiotic prescription patterns for 8 European countries (2010): Proportional prescription of antibiotic classes (ATC code). (DOCX)

S1 Table Comparison of antibiotics. (DOCX)

S2 Table Descriptive statistics of variables included in multilevel analysis. (DOCX)

S3 Table Resistance to 12 tested antibiotics in 8 European countries (%). (DOCX)

S4 Table Primary care practices and active patient population. (DOCX)
