# Supplementary material for: Antibiotic Exposure and Other Risk Factors for Antimicrobial Resistance in Nasal Commensal Staphylococcus aureus: An Ecological Study in 8 European Countries
Source: PLoS One. 2015 Aug 11;10(8):e0135094. doi: 10.1371/journal.pone.0135094 (PMC4532423; doi:10.1371/journal.pone.0135094)
Supplement: S1 Fig — (DOCX) [file pone.0135094.s001.docx]

# Supporting information 1

# Fig. Antibiotic prescription patterns for 8 European countries (2010): Proportional prescription of antibiotic classes (ATC code).
