# Supplementary material for: Antibiotic Exposure and Other Risk Factors for Antimicrobial Resistance in Nasal Commensal Staphylococcus aureus: An Ecological Study in 8 European Countries
Source: PLoS One. 2015 Aug 11;10(8):e0135094. doi: 10.1371/journal.pone.0135094 (PMC4532423; doi:10.1371/journal.pone.0135094)
Supplement: S1 Table — (DOCX) [file pone.0135094.s002.docx]

# Supporting information 1

# Table. Comparison of antibiotics

This table consists of all the antibiotic treatments in 2010 for each of the eight countries. Twelve tested substances are representative of several antibiotic classes.

**Table: Included antibiotic substances by class**

| **ATC3** | ATC5 code | Substance | Susceptibility test |
| --- | --- | --- | --- |
| **Tetracyclines (J01A)** | J01AA02 | Doxycycline | Tetracycline |
|  | J01AA04 | Lymecycline | Tetracycline |
|  | J01AA06 | Oxytetracycline | Tetracycline |
|  | J01AA07 | Tetracycline | Tetracycline |
|  | J01AA08 | Minocycline | Tetracycline |
| **Amphenicols (J01B)** | J01BA01 | Chloramphenicol | Not tested |
|  | J01BA02 | Thiamphenicol | Not tested |
|  | J01BA52 | Thiamphenicol, combinations | Not tested |
| **Penicillins (J01C)** | J01CA01 | Ampicillin | B-lact susc Peni |
|  | J01CA04 | Amoxicillin | B-lact susc Peni |
|  | J01CA08 | Pivmecillinam | Not tested |
|  | J01CE01 | Benzylpenicillin | B-lact susc Peni |
|  | J01CE02 | Phenoxymethylpenicillin | B-lact susc Peni |
|  | J01CE05 | Pheneticillin | B-lact susc Peni |
|  | J01CE06 | Penamecillin | B-lact susc Peni |
|  | J01CE07 | Clometocillin | B-lact susc Peni |
|  | J01CE08 | Benzathine benzylpenicillin | B-lact susc Peni |
|  | J01CE09 | Procaine benzylpenicillin | B-lact susc Peni |
|  | J01CE10 | Benzathine phenoxymethylpenicillin | B-lact susc Peni |
|  | J01CE30 | Combinations | B-lact susc Peni |
|  | J01CF02 | Cloxacillin | B-lact resist Peni |
|  | J01CF04 | Oxacillin | B-lact resist Peni |
|  | J01CF05 | Flucloxacillin | B-lact resist Peni |
|  | J01CR02 | Amoxicillin and enzyme inhibitor | B-lact resist Peni |
|  | J01CR04 | Sultamicillin | B-lact resist Peni |
| **Cephalosporins (J01D)** | J01DB01 | Cefalexin | Not tested |
|  | J01DB04 | Cefazolin | Not tested |
|  | J01DB05 | Cefadroxil | Not tested |
|  | J01DB07 | Cefatrizine | Not tested |
|  | J01DC02 | Cefuroxime | Not tested |
|  | J01DC04 | Cefaclor | Not tested |
|  | J01DC06 | Cefonicide | Not tested |
|  | J01DC07 | Cefotiam | Not tested |
|  | J01DC08 | Loracarbef | Not tested |
|  | J01DC10 | Cefprozil | Not tested |
|  | J01DD01 | Cefotaxime | Not tested |
|  | J01DD02 | Ceftazidime | Not tested |
|  | J01DD04 | Ceftriaxone | Not tested |
|  | J01DD08 | Cefixime | Not tested |
|  | J01DD13 | Cefpodoxime | Not tested |
|  | J01DD14 | Ceftibuten | Not tested |
|  | J01DD16 | Cefditoren | Not tested |
|  | J01DD54 | Ceftriaxone, combinations | Not tested |
|  | J01DE01 | Cefepime | Not tested |
|  | J01DF01 | Aztreonam | Not tested |
| **Sulfonamides & trimethoprim (J01E)** | J01EA01 | Trimethoprim | Co-trimoxazole |
|  | J01EB02 | Sulfamethizole | Not tested |
|  | J01EC01 | Sulfamethoxazole | Co-trimoxazole |
|  | J01EC02 | Sulfadiazine | Not tested |
|  | J01EE01 | Sulfamethoxazole and trimethoprim | Co-trimoxazole |
|  | J01EE03 | Sulfametrole and trimethoprim | Not tested |
| **Macrolides, lincosamides and streptogramins (J01F)** | J01FA01 | Erythromycin | Erythromycin |
|  | J01FA02 | Spiramycin | Erythromycin |
|  | J01FA06 | Roxithromycin | Erythromycin |
|  | J01FA07 | Josamycin | Erythromycin |
|  | J01FA09 | Clarithromycin | Erythromycin |
|  | J01FA10 | Azithromycin | Azithromycin |
|  | J01FA11 | Miocamycin | *Not tested* |
|  | J01FA13 | Dirithromycin | Not tested |
|  | J01FA15 | Telithromycin | Not tested |
|  | J01FF01 | Clindamycin | Clindamycin |
|  | J01FF02 | Lincomycin | Not tested |
|  | J01FG01 | Pristinamycin | Not tested |
| **Aminoglycosides (J01G)** | J01GA01 | Streptomycin | Not tested |
|  | J01GB01 | Tobramycin | Not tested |
|  | J01GB03 | Gentamicin | Gentamicin |
|  | J01GB06 | Amikacin | Not tested |
|  | J01GB07 | Netilmicin | Not tested |
| **Quinolone (J01M)** | J01MA01 | Ofloxacin | Ciprofloxacin |
|  | J01MA02 | Ciprofloxacin | Ciprofloxacin |
|  | J01MA03 | Pefloxacin | Ciprofloxacin |
|  | J01MA04 | Enoxacin | Ciprofloxacin |
|  | J01MA06 | Norfloxacin | Ciprofloxacin |
|  | J01MA07 | Lomefloxacin | Ciprofloxacin |
|  | J01MA12 | Levofloxacin | Ciprofloxacin |
|  | J01MA14 | Moxifloxacin | Not tested |
|  | J01MA17 | Prulifloxacin | Not tested |
|  | J01MB02 | Nalidixic acid | Not tested |
|  | J01MB04 | Pipemidic acid | Ciprofloxacin |
|  | J01MB07 | Flumequine | Not tested |
| **Combinations of antibacterials (J01R)** | J01RA02 | Sulfonamides, combinations | Not tested |
|  | J01RA04 | Spiramycin, combinations | Not tested |
| **Other antibacterials (J01X)** | J01XA01 | Vancomycin | Vancomycin |
|  | J01XB01 | Colistin | Not tested |
|  | J01XC01 | Fusidic acid | Not tested |
|  | J01XD01 | Metronidazole | Not tested |
|  | J01XE01 | Nitrofurantoin | Not tested |
|  | J01XE02 | Nifurtoinol | Not tested |
|  | J01XX01 | Fosfomycin | Not tested |
|  | J01XX03 | Clofoctol | Not tested |
|  | J01XX04 | Spectinomycin | Not tested |
|  | J01XX05 | Methenamine | Not tested |
|  | J01XX08 | Linezolid | Linezolid |
|  | J01XX09 | Daptomycin | Daptomycin |
