# Supplementary material for: Antibiotic Exposure and Other Risk Factors for Antimicrobial Resistance in Nasal Commensal Staphylococcus aureus: An Ecological Study in 8 European Countries
Source: PLoS One. 2015 Aug 11;10(8):e0135094. doi: 10.1371/journal.pone.0135094 (PMC4532423; doi:10.1371/journal.pone.0135094)
Supplement: S2 Table — (DOCX) [file pone.0135094.s003.docx]

# Supporting information 2

# Table. Descriptive statistics of variables included in multilevel analysis

|  | **Range** | **Average (N)** |
| --- | --- | --- |
| Total N = 6,062 |  |  |
| Age Patient | 4 - 106 | Average: 45,4 (s.e. 21,61)  Quartile 1: 4-29  Quartile 2: 30-46  Quartile 3: 47-62  Quartile 4: 63-106 |
| Gender Patient |  | % Male = 49 |
| Work in Healthcare |  | % Yes = 7.8 (N=398) |
| Work in Livestock |  | % Yes = 2.8 (N=146) |
| Work in Nursery |  | % Yes = 2.8 (N=147) |
| Living with children |  | % Yes = 17.0 (N=921) |
| Chronic skin condition |  | % Yes = 9,0 (N=546) |
| Number of GP visits  (last year) | 0, None;  1, Between 1-4 visits  2, 5 or more visits | % None = 8.5 (N=513)  % Between 1-4 = 58.1 (N=3521)  % 5 or more = 32.9 (N=1992) |
|  |  |  |
| Prescriptions Selected Total (per 100 active patients) 2010 | 1.3 – 184.6 | 40,02 (s.e. 31,64)  Quartile 1: 1,3-19,9  Quartile 2: 20,4-31.4  Quartile 3: 31.7-51  Quartile 4: 51.5-184.6 |
| % Penicillin 2010 | 2.4 – 66 % | Average = 30 % (s.e. 15%)  Quartile 1: 2.4-18.6  Quartile 2: 18.8-29.9  Quartile 3: 30.0-39.7  Quartile 4: 40.1-65.8 |
| MRSA |  | Total N = 78 |
| Multidrug resistant |  | Total N = 432 |
