# Supplementary material for: Antibiotic Exposure and Other Risk Factors for Antimicrobial Resistance in Nasal Commensal Staphylococcus aureus: An Ecological Study in 8 European Countries
Source: PLoS One. 2015 Aug 11;10(8):e0135094. doi: 10.1371/journal.pone.0135094 (PMC4532423; doi:10.1371/journal.pone.0135094)
Supplement: S3 Table — (DOCX) [file pone.0135094.s004.docx]

# Supporting information 3

# Table. Resistance to 12 tested antibiotics in 8 European countries (%)

|  | ***Austria*** | ***Belgium*** | ***Croatia*** | ***France*** | ***Hungary*** | ***Netherlands*** | ***Spain*** | ***Sweden*** |
| --- | --- | --- | --- | --- | --- | --- | --- | --- |
| **Tetracyclines (J01AA)** | **3.5** | **4.5** | **7.2** | **2.9** | **6.7** | **4.5** | **1.8** | **2.7** |
| **Beta-lactamase susceptible Penicillins**  **(J01CA / J01CE)** | **64.8** | **72.0** | **77.9** | **74.9** | **76.1** | **68.8** | **87.1** | **65.3** |
| **Beta-lactamase resistant Penicillins**  **(J01CF / J01CR)** | **1.5** | **2.1** | **2.0** | **1.7** | **1.5** | **0.8** | **1.2** | **0.0** |
| **Sulfonamides and Trimethoprim (J01E)** | **0.2** | **0.3** | **0.4** | **0.5** | **0.4** | **0.2** | **0.3** | **0.0** |
| **Macrolides (J01FA)** | **13.4** | **16.3** | **5.7** | **16.5** | **12.1** | **5.3** | **11.2** | **1.5** |
| **Macrolides – Azithromycin (J01FA10)** | **13.6** | **16.3** | **5.7** | **16.9** | **12.1** | **6.7** | **11.6** | **1.5** |
| **Lincosamides (J01FF)** | **11.2** | **14.6** | **5.3** | **14.3** | **12.1** | **4.9** | **9.6** | **1.4** |
| **Aminoglycosides (J01GB)** | **2.0** | **0.5** | **2.0** | **0.0** | **0.0** | **0.7** | **1.0** | **0.0** |
| **Quinolones (J01M)** | **2.5** | **2.4** | **1.2** | **1.9** | **2.0** | **2.0** | **2.3** | **0.6** |
| **Vancomycin (J01XA01)** | **0.0** | **0.0** | **0.0** | **0.0** | **0.0** | **0.0** | **0.0** | **0.0** |
| **Linezolid (J01XX08)** | **0.0** | **0.0** | **0.0** | **0.0** | **0.0** | **0.0** | **0.0** | **0.0** |
| **Daptomycin (J01XX09)** | **0.0** | **0.0** | **0.0** | **0.0** | **0.0** | **0.2** | **0.0** | **0.0** |
