# Supplementary material for: Antibiotic Exposure and Other Risk Factors for Antimicrobial Resistance in Nasal Commensal Staphylococcus aureus: An Ecological Study in 8 European Countries
Source: PLoS One. 2015 Aug 11;10(8):e0135094. doi: 10.1371/journal.pone.0135094 (PMC4532423; doi:10.1371/journal.pone.0135094)
Supplement: S4 Table — (DOCX) [file pone.0135094.s005.docx]

# Supporting information 4

# Table. Primary care practices and active patient population

| **Country** | **N practices** | **N active patients** |
| --- | --- | --- |
| **Austria** | 20 | 58,156 |
| **Belgium** | 18 | 38,755 |
| **Croatia** | 20 | 33,237 |
| **France** | 17 | 64,252 |
| **Hungary** | 20 | 30,679 |
| **Spain** | 38 | 637,999 |
| **Sweden** | 10 | 81,469 |
| **The Netherlands** | 21 | 79,517 |
| **Total** | **164** | **1,024,064** |
